# Viscoplastic Modeling of Surface Relief Grating Growth on Isotropic and Preoriented Azopolymer Films

**DOI:** 10.3390/polym15020463

**Published:** 2023-01-16

**Authors:** Nina Tverdokhleb, Sarah Loebner, Bharti Yadav, Svetlana Santer, Marina Saphiannikova

**Affiliations:** 1 Institute Theory of Polymers, Leibniz Institute of Polymer Research Dresden, 01069 Dresden, Germany; 2 Institute of Physics and Astronomy, University of Potsdam, 14476 Potsdam, Germany

**Keywords:** side-chain azopolymer, photoinduced deformation, finite element modeling, photosoftening effect, orientation approach

## Abstract

We report on solving of two intriguing issues concerning the inscription of surface relief gratings within azopolymer thin films under irradiation with SS, PP and RL interference patterns. For this, we utilize the orientation approach and viscoplastic modeling in combination with experimental results, where the change in surface topography is acquired in situ during irradiation with modulated light. First, the initial orientation state of polymer backbones is proved to be responsible for the contradictory experimental reports on the efficiency of the SS interference pattern. Different orientation states can influence not only the phase of SS grating but also its height, which is experimentally confirmed by using special pretreatments. Second, the faster growth of gratings inscribed by the RL interference pattern is shown to be promoted by a weak photosoftening effect. Overall, the modeled results are in good agreement with the order of relative growth efficiency: RL–PP–SS.

## 1. Introduction

Polymers that can change their molecular architecture under such external stimulation as light have been of great interest for the scientific community during the last quarter of the century. Particularly known are photosensitive azopolymers, which have many applications in different fields due to their high versatility. They can be used in electronics and photonics [1,2,3], biology [4,5,6,7], medicine [8,9], liquid crystalline technology [10,11], modern soft robotics [12,13,14], etc. Furthermore, the light-responsive azosubstances can play a role of molecular switches [15,16,17,18] and fancy molecular motors [19,20,21] in different processes and devices, for instance with the aim to control surface wettability [22,23,24,25]. Some natural phenomena, such as fly-trapping plants [26], can be mimicked by designing microrobotic systems incorporating azobenzene chromophores.

In modern engineering, a special interest has been evoked by a unique opportunity to control the deformations of thin azopolymer films depending on the interference pattern (IP) of light beams [27,28,29,30,31]. This phenomenon of optically induced mass transport with the appearance of periodical topographical structures (surface relief gratings, or SRGs) was first recorded in the middle of the 1990s [32,33]. Azobenzene groups, covalently attached to the polymer backbones, are thought to be responsible for the topographical restructuring due to their cyclic *trans-cis* photoswitching ability and light-induced orientation. To support the experimental findings, multiple theoretical interpretations of this phenomenon have been developed, but only a few of them have considered the features of underlying molecular architecture. Moreover, some peculiar experimental observations of photoinduced deformations in azopolymer samples remain unexplained and are still discussed [22,27].

Most likely, the photoinduced deformations can be explained by the direct transformation of the light energy into the mechanical one. For this purpose, over the years, the orientation approach has been elaborated [34,35,36], which establishes a clear relation between the light characteristics and the molecular and mechanical properties of azo-containing materials. Azobenzene chromophores tend to be oriented perpendicular to the electric field vector ***E*** under the influence of polarized light [37]. In molecular glasses [38,39], the reorientation of the chromophores results in negative mechanical stress and thus in the uniaxial contraction of the material along the polarization direction. This explains the inscription of SRGs in molecular glasses, as we showed in [34,40]. In side-chain azopolymers, the reorientation of the azobenzene groups perpendicular to ***E*** induces the reorientation of the main chains along ***E***. Both processes can be described with the help of effective orientation potentials [35,41]. The local ordering of the polymer backbones within thin film generates corresponding mechanical stress that causes plastic deformations in the glassy polymer, with the appearance of different grating structures determined by the light intensity and polarization distribution [42,43].

In the previous study, we successfully implemented the orientation approach to model the inscription of SRGs in the pre-elongated photosensitive colloids under irradiation with SS and PP intensity interference patterns (IIPs), as well as the restructuring of film edges by RL and LR polarization interference patterns (PIPs) [43]. A striking resemblance between the experimentally recorded and modeled structures was established. This motivated us to approach two unresolved issues regarding the inscription of SRGs on the thin azopolymer films. The first question is, why are the experimental reports so contradictory about the efficiency of the SS intensity pattern? While some groups found that this pattern did not inscribe any grating [44,45,46,47], others observed a modest efficiency [48,49,50]. Interestingly, the optical gradient theory [51] predicts that SS gratings cannot be inscribed at all, because of a zero force in the direction of the grating vector. Therefore, it would be important to identify the factors that could promote the emergence of SS topography. The second question is, why are the gratings produced by PIPs usually higher than those produced by IIPs [44,50,52]? Previous theories predicted an opposite tendency for RL IPs and PP IPs [53,54].

## 2. Materials and Methods

Poly[1-[4-(3-carboxy-4-hydroxyphenylazo)benzenesulfonamido]-1,2-ethanediyl, sodium salt] (PAZO) and poly[(methyl methacrylate)-co-(Disperse Red 1 acrylate)] (pDR1) were purchased from Sigma-Aldrich (Darmstadt, Germany). The polymer films of PAZO are prepared by spin casting 100 μL of the polymer solution (170 mg PAZO dissolved in a 1 mL solution containing a mixture of 95% methoxyethanol and 5% ethylene glycol) on thin glass slides at 3000 rpm for 1 minute. The pDR1 polymer films are casted at the same conditions from a chloroform solution of 60 mg/mL concentration. The film thickness is measured using the NTEGRA (NT-MDT, Moscow, Russia) atomic force microscope (AFM) cross-section analysis of a scratch within the polymer film.

The interference pattern is generated using a homemade two-beam interferometer with a continuous wave diode pumped solid state laser of a 491 nm wavelength (Cobolt Calypso, Cobolt, Solna, Sweden), as described in details in [55]. In short, the two-beam interference irradiation is performed using three interference patterns: SS and PP (intensity interference pattern) and RL (polarization interference pattern). The beam diameter is set to 4 mm for the pump beams; the intensity is set to 100 mW/cm^2^, and a beam splitter, followed by a half-wave plate and a polarizer per beam, is added to generate two beams of the same intensity. The in situ AFM measurements are performed using a PicoScan (Molecular Imaging), working in intermittent contact mode. The scan speed is 1 Hz, with a scan-area of 10 × 10 μm with 512 × 512 pixels. Commercial tips (Nanoworld-Point probe, Neuchâtel, Switzerland) with a resonance frequency of 130 kHz and a spring constant of 15 N/m are used for measurements.

The AFM measurements performed ex situ (measurements of film thickness and the shape of the gap) are carried out using an NTEGRA (NT-MDT) AFM operating in intermittent mode. Commercial tips (Nanoworld-Point probe, Neuchâtel, Switzerland) with a resonance frequency of 320 kHz and a spring constant of 42 Nm are used for these measurements. All experiments are carried out under yellow light in the laboratory (to avoid undesirable photoisomerization) and under ambient conditions, i.e., at room temperature with a relative humidity of 55%.

### Theoretical Approach

To solve the questions posed in the introduction, we model the dynamical growth of surface relief gratings induced by the PP, SS and RL interference patterns using the orientation approach. This approach defines the light-induced stress τ via the rate of change of the 2nd-order orientation tensor ⟨uu⟩ of rigid backbone segments [41]:

(1) $$\tau =3nkT\lambda \frac{\partial }{\partial t}\langle uu\rangle$$

where n is the number density of segments; λ is their rotational time in the absence of light; and u is the unit orientation vector of the segment. The tensors ⟨uu⟩ and τ are diagonal owing to the axial symmetry around the light polarization ***E*** [41]. The diagonal components of ⟨uu⟩ can be calculated as follows:

(2) $$\frac{\partial }{\partial t}\langle u_{∥}^{2}\rangle =\frac{1}{\lambda }\left[V_{r}(\langle u_{∥}^{2}\rangle -1)\langle u_{∥}^{2}\rangle -\langle u_{∥}^{2}\rangle +\frac{1}{3}\right]\;\frac{\partial }{\partial t}\langle u_{⊥}^{2}\rangle =\frac{1}{\lambda }\left[(V_{r}\langle u_{∥}^{2}\rangle -1)\langle u_{⊥}^{2}\rangle +\frac{1}{3}\right]$$

where $\left\langle u_{∥}^{2}\right\rangle$ and $\left\langle u_{⊥}^{2}\right\rangle$ are components of the orientation tensor parallel and orthogonal to the electric field vector ***E***. In our latest paper [43], Equation (2) is solved for different IPs, starting from isotropic and anisotropic initial states. The parameter $V_{r}=2qmV_{0}/5kT$ defines the reduced strength of the effective orientation potential, which acts on the polymer backbones during illumination [41]. Here, $V_{0}$ is the strength of the effective orientation potential acting on the azobenzene chromophores. Detailed information on these potentials can be found elsewhere [35,41]. The molecular architecture of an azopolymer is described with the help of two parameters: m is the number of azobenzene groups attached to one backbone segment, and q is the shape factor, the value of which depends on the art of the attachment. It may vary between q=−0.5 for azobenzenes attached perpendicular to the polymer segment and q=1 for azobenzenes attached parallel to the polymer segment or incorporated directly into the main chain.

The viscoplastic modeling of topographical structures is performed using the finite element software ANSYS. In particular, the Perzyna model is applied, which prescribes the relation between the rate of plastic strain ${\dot{\varepsilon }}_{pl}$ and the magnitude of light-induced stress $\tau_{eq}$:

(3) $${\dot{\varepsilon }}_{pl}=\gamma \left(\frac{\tau_{eq}}{\tau_{yield}}-1\right)$$

where $\tau_{yield}$ is the yield stress and $\gamma =\tau_{yield}/\left(3\eta \right)$ regulates the viscosity of plastic flow η. We use the following parameters to describe the material properties of an amorphous azopolymer [41,42]: $\tau_{yield}=$ 10 MPa, γ= 0.01 s^−1^ and λ= 1000 s; the reduced strength $V_{r}=$−36.2. This corresponds to the initial light-induced stress $\tau_{xx,0}=-\frac{2nkTV_{r}}{3}=\;$25 MPa if the number density of polymer segments n= 2.5 × 10^26^ m^−3^. Note a time rescaling: 1 s in the modeling roughly corresponds to 1 min of experiment.

In the experiment, the atomic force microscope (AFM) acquires a central part of the film. Therefore, to compare the efficiency of different IPs, we model a parallelepiped unit cell of an infinite sample. The *z*-axis is orthogonal to the xy-plane of the substrate, where the *x*-axis lies along the grating vector direction. Details on boundary conditions (BC) are given in the Supplementary Materials (SM). As we show bellow, BCs in the y-direction crucially affect the appearance of the inscribed surface patterns. For the finite element realization of IIPs, linearly polarized light with sinusoidally varying intensity is applied to the modeled samples:

(4) $$I\left(x\right)=2I_{0}\cos^{2}\left(\frac{\pi x}{D}\right)$$

where $I_{0}$ is the intensity of the laser beam and *D* the grating period. In RL and LR polarization patterns, the light has constant intensity $I\left(x\right)=I_{0}$, and its linear polarization rotates along the grating period. Therefore, the coordinate system of each finite element is rotated in respect to the laboratory system, as described elsewhere [43].

## 3. Results

**Figure 1:**
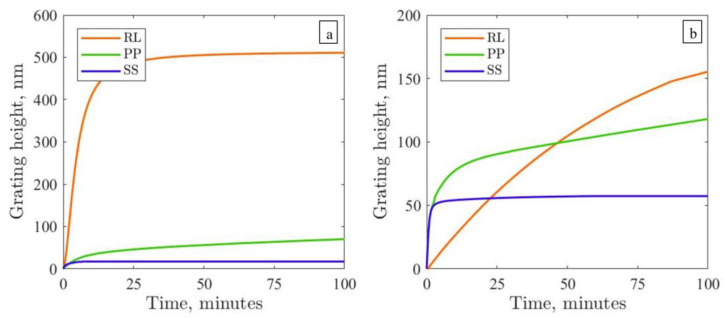
Experimentally recorded time-dependent growth of SS, PP and RL gratings for (**a**) pDR1 films of 500 nm thickness and (**b**) PAZO films of 1 µm thickness. The irradiation intensity in all cases is 100 mW/cm^2^, as measured at the sample; the irradiation wavelength is 491 nm; and the optical grating period is 2 µm.

Let us explore the first problem of SS grating inscription. Here, the electric field vector ***E*** points along the y-axis. Our modeling shows that no surface changes appear under SS IP if the initial state of polymer backbones is isotropic (see Figures S1 and S2). This is in good agreement with the experimental data for the Azo-Psi and Azo-PCMS polymers; see in [50]. Even with free BCs, when the azopolymer is allowed to move along the light polarization ***E*** without limitations, the grating height is merely 2 nm. However, depending on the polymer (at a fixed film height, intensity and wavelength of irradiation and optical period), the height of the SRG gratings inscribed during irradiation with SS IP broadly varies. In the present study, we measured 20 nm for pDR1 and 50 nm for the PAZO polymers (see the blue lines in Figure 1).

As for irradiation, two parameters should influence the inscription of topographical structures: the absorption of light by the azogroups and the Gaussian distribution of light intensity in the illuminated spot. Absorption decreases the light intensity in the sample depth and, hence, the light-induced stress tensor. So we observe that this factor only further diminishes the grating height with free BCs along the y-axis. More probably, the growth of the SS gratings can be enhanced when the Gaussian distribution of light intensity is accounted for. This assumption has been thoroughly checked in extended modeling. As reported in the Supplementary Materials, only the extreme focusing of the laser beam (down to 1 μm vs. 2 mm radius in the experiment) is able to enhance the surface deformations under the SS interference pattern, which then lose their sinusoidal form.

**Figure 2:**
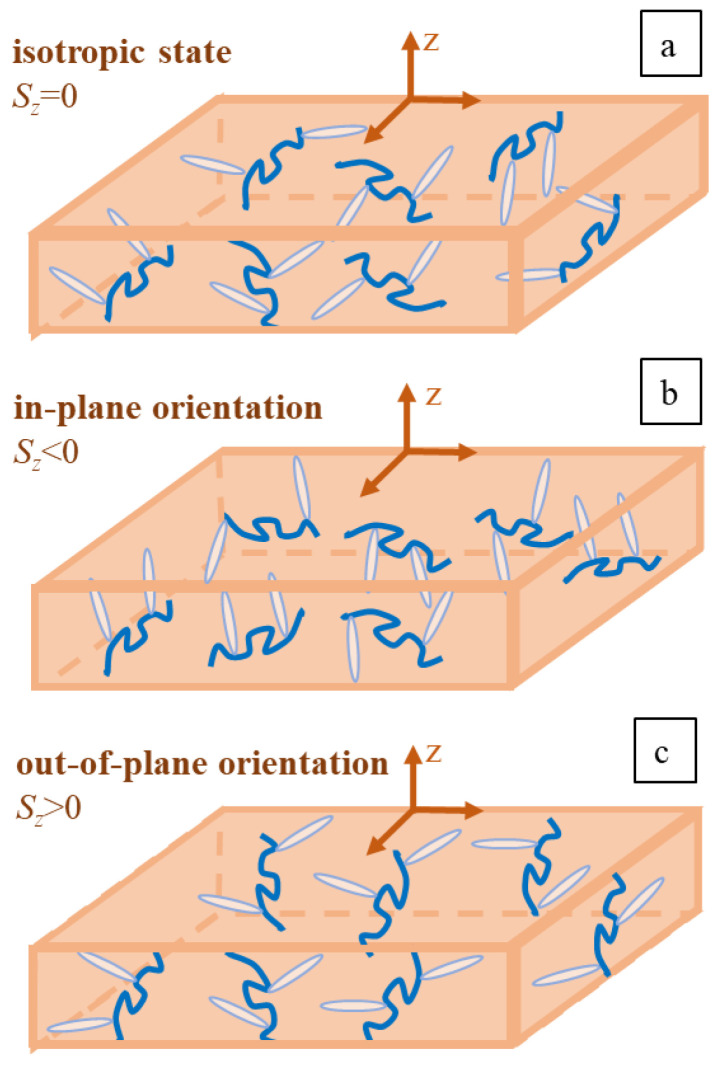
Three cases of initial polymer backbone orientations: (**a**) isotropic state—backbones are without preorientation; (**b**) case 1—backbones have in-plane orientation; and (**c**) case 2 corresponds to the out-of-plane backbone orientation. $S_{z}$ is the order parameter of polymer backbones in respect to the *z*-axis.

**Figure 3:**
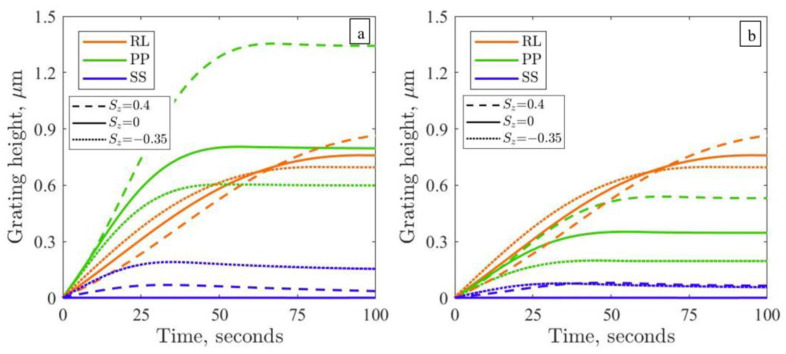
Modeled time-dependent growth of SS, PP and RL gratings at periodic BCs along the *y*-axis for different initial backbone orientations: (**a**) in the absence ($\gamma =\gamma_{0}=$ 0.01 s^−1^) and (**b**) presence of photosoftening (γ is given by Equation (5)). The film thickness is 1 µm. The initial light-induced stress is 25 MPa.

Thus, it still remains unclear why the SS gratings can be inscribed in the experiment, whereas the modeling predicts the absence of this type of grating for isotropic polymer films (Figure 2a). Under such initial conditions, it is also not possible to explain why in the experiments the gratings produced by the RL polarization pattern are usually higher than those produced by the PP intensity pattern [44,50]; see, for example, the growth curves for pDR1 and PAZO measured in the present study (Figure 1). The modeling shows that the growth rate of the PP grating is twice as large as that of the RL grating (green vs. red solid lines in Figure 3a). However, plastic deformations saturate earlier for PP IP, and thereby, the height of the RL grating approaches the height of the PP grating at larger inscription times.

With the increase in the grating period *D* = 1, 2, 4 μm, both patterns become higher, but the relation between them stays unchanged (see Figure S4). To shed light on this issue, we decided to check the following hypothesis: *What if the process of film preparation causes a preferential orientation of polymer backbones inside a sample?* The glass substrate is spin coated with azopolymer solution and rotated at a high speed around the z-axis. It is plausible to assume that such a procedure creates an axially symmetric orientation state, for which the x- and y-components of the orientation tensor <***uu***> are equal to each other. The degree of backbone orientation in respect to the z-axis is characterized by the order parameter $S_{z}=\frac{3}{2}\langle u_{z}^{2}\rangle -\frac{1}{2}$. Given this assumption, two cases can be distinguished (Figure 2):

1. Polymer backbones tend to lie in the plane of substrate, $\langle u_{z}^{2}\rangle <\langle u_{x}^{2}\rangle =\langle u_{y}^{2}\rangle ,\;S_{z}<0$.
2. Polymer backbones have a tendency to align along the *z*-axis, $\langle u_{z}^{2}\rangle >\langle u_{x}^{2}\rangle =\langle u_{y}^{2}\rangle ,\;S_{z}>0$.

We model irradiation with all three IPs for case 1, choosing $\left\langle u_{z}^{2}\right\rangle$ = 0.1 and hence $S_{z}$= –0.35 (see the dotted lines in Figure 3). The crucial result here is the emergence of the SS grating with a height of approximately 100 nm. Because of anisotropic initial state, the light-induced stress tensor becomes asymmetric around the *z*-axis, and this promotes the inscription of the SS gratings even at periodic BCs along the *y*-axis. At a larger inscription time, we observe a noticeable decrease in the PP grating height in comparison with the isotropic state (compare the dotted and solid green lines in Figure 3); as a result, the RL grating slightly exceeds the PP one at the end of the inscription. Additionally, we perform calculations for case 2, choosing $\langle u_{z}^{2}\rangle =0.6$ and hence $S_{z}$ = 0.4. The height of the PP grating is considerably enhanced in respect to the isotropic state, while the growth of the RL grating has only marginally improved (compare the broken and solid lines in Figure 3a). With this, the relation between the height of the intensity and the polarization gratings starts to contradict the experiment (Figure 1). Moreover, the height of the SS grating noticeably decreases: two and half times compared to case 1 (see the dotted lines in Figure 3a). Hence, at first glance, case 2 is less favorable than case 1.

We show that by accounting for the initial preorientation of the polymer backbones, it is possible to explain the inscription of the SS grating and to interpret the difference in the growth of the PP and RL gratings. Nevertheless, the experimentally observed relation between the grating growth for the PP and RL interference patterns remains hidden if only the initial orientation state is adjusted. At this point, it is worth checking an additional hypothesis: *What if inhomogeneous irradiation modulates the local viscosity of the azopolymer?*

There are indirect experimental indications that light of a moderate intensity (up to 200 mW/cm^2^) can decrease the viscosity of glassy polymers up to 1 order of magnitude [40,56]. Maybe, this photosoftening effect also controls the grating growth and defines the difference in SRG’s appearance between the intensity and the polarization patterns. The main reason here is that in the PP and SS patterns, the intensity of the light sinusoidally changes along the grating period; see Equation (4). Hence, the strongly illuminated stripes with maximal intensity *I*_max_ = 2*I*_0_ are followed by unilluminated stripes *I*_min_ = 0 in the film irradiated with intensity patterns. In PIPs, the light intensity *I*_0_ stays unchanged along the grating period. We assume that this distinction determines the local dependence of plastic flow viscosity *η* on light intensity. As we described above, *η* is inversely proportional to the parameter γ in the Perzyna model; see Equation (3). When the light irradiation decreases the viscosity of the azopolymer, γ should increase. We use Equation (5) to implement the light-induced change of γ:

(5) $$\gamma =\gamma_{0}e^{\left(\frac{V\left(x\right)}{V_{r,0}}-1\right)}=\gamma_{0}e^{\left(2cos^{2}\left(\frac{\pi x}{D}\right)-1\right)}$$

where the reduced strength of the orientation potential $V\left(x\right)=V_{r,0}$ for PIPs and $V\left(x\right)=2V_{r,0}cos^{2}\left(\frac{\pi x}{D}\right)$ for IIPs. It is assumed that a noticeable decrease in viscosity and, hence, an increase in γ starts at $V_{r,0}$, which corresponds to the impact of light with the intensity *I*_0._ For such a choice, the parameter γ changes with intensity $I=0\ldots I_{0}\ldots 2I_{0}$ as follows: $\gamma =\frac{\gamma_{0}}{e}\ldots \gamma_{0}\ldots \gamma_{0}e$. Accordingly, the viscosity in various regions of IIPs is changed in *e*^2^ times, while for PIPs, the viscosity is constant.

The results of viscoplastic modeling for a photosoftened azopolymer are presented in Figure 3b. Accounting for the regions with different viscosities in IIPs considerably reduces the height of the corresponding gratings. In particular, for the PP gratings inscribed from the initial isotropic orientation, the height decreases more than two times (compare the solid green lines in Figure 3). The growth rate of the PP grating becomes slower than that of the RL grating, the latter being unaffected by the change in viscosity. With this, the modeling results for the initial isotropic state resemble the growth of the SS, PP and RL gratings inscribed on the pDR1 and PAZO polymers (Figure 1). Interestingly, the SS gratings grow in a similar manner from two anisotropic states for a photosoftened polymer (compare the dotted and broken lines in Figure 3b), reaching a height of 50 nm.

## 4. Discussion

Until now, we had considered only the dynamics of grating growth under different conditions. Several efforts have previously been made to correlate the phase of gratings with the phase of interference patterns. The most thorough assignment is reported in ref. [49], with a single correction for the RL pattern made later in ref. [55]. To be able to solve the problem of grating inscription, it is necessary to check our modeling predictions against this assignment experiment. The phases of the modeled PP and the RL gratings match the assignment; see Figure S5. The valleys in the PP grating correspond to the maximal intensity of light (Figure S1a) because the azopolymer is stretched along *E*. Switching from periodic BCs to free BCs along the y-direction slightly decreases the grating growth because some material protrudes symmetrically at the hill positions (intensity minima) into free space. In the RL grating, the hills grow at positions where *E* is perpendicular to the grating vector. Interestingly, modeling with periodic BCs results in the antisymmetric modulation of film edges along the y-axis (Figure S5c,d). As reported in our previous paper [43], the free BCs lead to the appearance of differently structured edges: spiky protrusions from one side and fan-like protrusions from the other side (Figure S1c). Such shape is formed due to the stretching of the material elements in different directions by the light with rotating linear polarization. Therefore, at the edge with converging *E* vectors, the material is expelled out of the film, while at the edge with diverging *E* vectors, the material is pushed inside the film.

**Figure 4:**
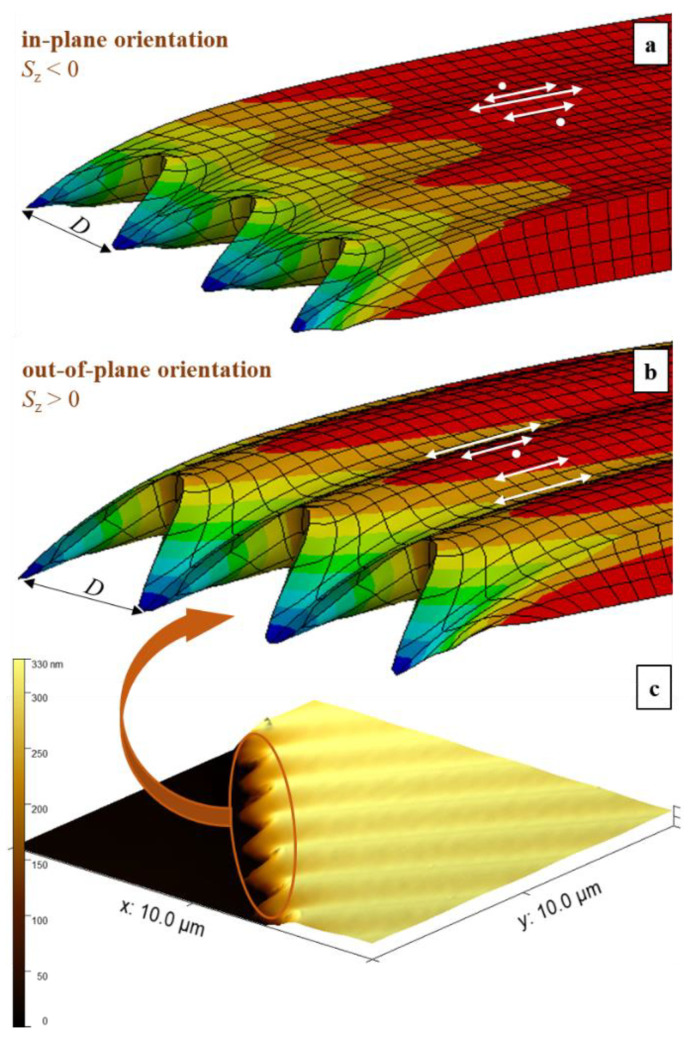
Modeled SS gratings inscribed on the 1 µm thick film after 25 s at free BCs along the polarization direction for two initial backbone orientations: (**a**) case 1 and (**b**) case 2. The local distribution of the electric field vector is depicted by white arrows. The initial light-induced stress is 25 MPa. (**c**) AFM micrograph of the 300 nm thick pDR1 polymer film irradiated with SS IP for 1 h. The polymer film is scratched before irradiation to allow for the boundary deformation.

As we discussed above, the SS grating cannot be inscribed from the isotropic initial state at any of the BCs (Figures S1b and S2b; Video S1). Let’s look at the appearance of this grating in case 1 (Figure 2b) with the preferential orientation of backbones in the plane of the substrate (azobenzenes are aligned perpendicular to the substrate). The maximal intensity of light corresponds to the hills in the SS grating, for both free and periodic BCs (Figure 4a and Figure S5e; Video S2). At free BCs, the protrusions of free edges appear at the hill positions in the bulk of the SS grating. In case 2 (Figure 2c), with the backbones aligned along z-axis (azobenzenes prefer to align in the plane of substrate), the maximal intensity of light corresponds to the valleys in the SS grating (Figure 4b and Figure S5f; Video S3), which is in accordance with the assignment in ref. [49]. The protrusions of free edges appear at the valley positions in the bulk of the SS grating. This agrees well with the AFM micrograph for the scratched pDR1 film (Figure 4c). Hence, we found that the initial preorientation does not affect the phase of the PP and RL gratings but can change the phase of the SS grating (Figure S5e,f).

To better understand the influence of the initial orientation, we treated the scratched azopolymer films with different stimuli, specifically heat, linearly (P) polarized light and circularly (R) polarized light, before the exposure to the SS IP. The results are summarized in Table 1. The pDR1 film, used as prepared, allows us to inscribe the SS grating with a height of 30 nm. Small protrusions appear at the valley positions, which indicates the preferable orientation of the azobenzenes in the plane of the substrate. Half an hour of heating at 120 °C results in an almost flat film (∆*h* ≈ 2 nm), which is expected, as the heating above the pDR1 glass transition (*T*_g_ = 100 °C) induces the isotropic orientation of the azobenzenes and the main chains. Pretreatment with P or R polarized light with an intensity of 100 mW/cm^2^ leads to the same result. This means that during the pre irradiation, the azobenzenes are oriented out of the substrate plane, which results in the in-plane orientation of the backbones. Circularly polarized light with the much smaller intensity of 10 mW/cm^2^ seems unable to fully destroy the initial orientation.

Very interesting results are found for the PAZO films (Table 1). Here, the preheating does not prohibit the inscription of the SS grating of the height ∆*h* ~ 70 nm. A small difference between the as-prepared and the preheated samples can be explained by the larger thickness of the latter. It is known from a previous study [55] that the PAZO polymer does not exhibit a glass transition, and as a result, the films made from it do not respond on preheating. However, the treatment with P and R polarized light appears to be quite effective in destroying the initial orientation of azobenzenes, as indicated by the drop in the SS height to 10–20 nm. Altogether, the modeling results and the data shown in Table 1 support our working hypothesis that the appearance of the SS grating and its efficiency crucially depend on the initial orientation state of the side-chain azopolymer used in a particular experiment. This would explain contradictory reports in the literature [44,45,46,47,48,49,50].

## 5. Conclusions

In this study, we presented the theoretical solution for two problems in the inscription of SRGs during irradiation with the RL, PP and SS interference patterns. For the first time, a possible reason for the contradictory experimental data for the SS gratings was identified as the initial orientation of the polymer backbones. By changing the order parameter $S_{z}$, which describes the degree of backbone preorientation in respect to the *z*-axis, it became possible to control not only the height of the SS grating but also its phase. To solve the second problem, we assumed a weak photosoftening effect with a constant reduction in the plastic flow viscosity for PIPs and its sinusoidal variation for IIPs. With such an approach, the relation of the gratings’ dynamical growth between the RL, PP and SS patterns was explained to be in agreement with numerous experimental data.

## Figures and Tables

**Table 1:** The height of SS grating ∆*h* inscribed on pDR1 and PAZO after different pretreatments.

Thickness, nm	Pretreatment	Height of SS Grating, nm
**pDR1**		
300	as prepared	30
	heated 120 °C, 30 min	2
	R 100 mW/cm^2^, 10 min	2
	P 100 mW/cm^2^, 10 min	2
	R 10 mW/cm^2^, 10 min	8
**PAZO**		
740	as prepared	65
810	heated 120 °C, 1 h	74
740	R 100 mW/cm^2^, 1 h	10
810	P 100 mW/cm^2^, 1 h	19
810	R 10 mW/cm^2^, 1 h	12

## Data Availability

On inquiry, the data presented in this study are available from the authors.

## Supplementary Materials

The following supporting information can be downloaded at https://www.mdpi.com/article/10.3390/polym15020463/s1. The supporting information consists of the next sections: the inscription of the surface relief gratings on an initially isotropic sample; influence of boundary conditions; impact of Gaussian intensity distribution; influence of the grating period *D* on the grating growth; and influence of initial orientation on phases of gratings. List of videos: Video S1: Plastic deformation of the azopolymer film under irradiation with SS interference pattern from the initial isotropic state; Video S2: Plastic deformation of the azopolymer film under irradiation with SS interference pattern from the in-plane orientation of polymer backbones (case 1); Video S3: Plastic deformation of the azopolymer film under irradiation with SS interference pattern from the out-of-plane orientation of polymer backbones (case 2). Figure S1: Modeled gratings with free BCs in y-direction after 100 s of inscription with PP, SS and RL interference patterns. Initial isotropic state. Colors correspond to directional deformations along z-axis: from maximal stretching (red) to maximal compression (blue). Figure S2: Modeled gratings with Frictionless support in y-direction after 100 s of inscription with PP, SS and RL interference patterns. Initial isotropic state. Colors correspond to directional deformations along z-axis: from maximal stretching (red) to maximal compression (blue). Figure S3: Modeled gratings with Frictionless support in y-direction after 100 s of inscription by differently focused Gaussian beams: (a) SS, ω = 1 μm, (b) SS, ω = 10 μm, (c) PP, ω = 5 μm, (d) RL, ω = 5 μm. The size of the sample in a, c and d is 4 × 10 × 1 μm^3^, for b the size is taken 4 × 20 × 1 μm^3^ to accommodate a larger irradiation spot. Initial isotropic state. Figure S4. Time-dependent growth of PP and RL gratings at different optical periods: D = 1, 2 and 4 μm: Initial isotropic state. Note that the height of both gratings increases with the grating period. Figure S5: Modeled gratings for two initial anisotropic cases discussed in the main text. a, c, e—case 1 and b, d, f—case 2. Infinite sample with periodic BCs in y-direction has the unit cell 4 × 2 × 1 μm^3^. Note that the phase of PP and RL gratings is not influenced by the initial orientation state, whereas the phase of SS grating flips: the maximal stretching along z-axis (red hills) grow either at maximal or minimal intensity of light.

## References

[1] Stracke A., Wendorff J.H., Goldmann D., Janietz D., Stiller B. (2000). Gain effects in optical storage: Thermal induction of a surface relief grating in a smectic liquid crystal. Adv. Mater..

[2] Natansohn A., Rochon P. (1999). Photoinduced motions in azobenzene-based amorphous polymers: Possible photonic devices. Adv. Mater..

[3] Oscurato S.L., Reda F., Salvatore M., Borbone F., Maddalena P., Ambrosio A. (2021). Large-Scale Multiplexed Azopolymer Gratings with Engineered Diffraction Behavior. Adv. Mater. Interfaces.

[4] Shimoboji T., Larenas E., Fowler T., Kulkarni S., Hoffman A.S., Stayton P.S. (2002). Photoresponsive polymer-enzyme switches. Proc. Natl. Acad. Sci. USA.

[5] Banghart M., Borges K., Isacoff E., Trauner D., Kramer R.H. (2004). Light-activated ion channels for remote control of neuronal firing. Nat. Neurosci..

[6] Goulet-Hanssens A., Barrett C.J. (2013). Photo-control of biological systems with azobenzene polymers. J. Polym. Sci. Part A Polym. Chem..

[7] Konrad D.B., Frank J.A., Trauner D. (2016). Synthesis of Redshifted Azobenzene Photoswitches by Late-Stage Functionalization. Chemistry.

[8] Zakrevskyy Y., Cywinski P., Cywinska M., Paasche J., Lomadze N., Reich O., Löhmannsröben H.-G., Santer S. (2014). Interaction of photosensitive surfactant with DNA and poly acrylic acid. J. Chem. Phys..

[9] Schimka S., Santer S., Mujkic-Ninnemann N.M., Bleger D., Hartmann L., Wehle M., Lipowsky R., Santer M. (2016). Photosensitive Peptidomimetic for Light-Controlled, Reversible DNA Compaction. Biomacromolecules.

[10] Chigrinov V.G., Kozenkov V.M., Kwok H.-S. (2008). Photoalignment of Liquid Crystalline Materials: Physics and Applications.

[11] Tabiryan N.V., Roberts D.E., Liao Z., Hwang J.-Y., Moran M., Ouskova O., Pshenichnyi A., Sigley J., Tabirian A., Vergara R. (2021). Advances in Transparent Planar Optics: Enabling Large Aperture, Ultrathin Lenses. Adv. Opt. Mater..

[12] Yu Y.L., Nakano M., Ikeda T. (2003). Directed bending of a polymer film by light-Miniaturizing a simple photomechanical system could expand its range of applications. Nature.

[13] Ryabchun A., Bobrovsky A. (2019). Photocontrollable Deformations of Polymer Particles in Elastic Matrix. Adv. Opt. Mater..

[14] Lancia F., Ryabchun A., Nguindjel A.-D., Kwangmettatam S., Katsonis N. (2019). Mechanical adaptability of artificial muscles from nanoscale molecular action. Nat. Commun..

[15] Bléger D., Hecht S. (2015). Visible-Light-Activated Molecular Switches. Angew. Chem. Int. Ed..

[16] Bléger D. (2016). Orchestrating Molecular Motion with Light – From Single (macro)Molecules to Materials. Macromol. Chem. Phys..

[17] Koch M., Saphiannikova M., Guskova O. (2021). Cyclic Photoisomerization of Azobenzene in Atomistic Simulations: Modeling the Effect of Light on Columnar Aggregates of Azo Stars. Molecules.

[18] Koch M. (2022). The Influence of Light on a Three-Arm Azobenzene Star: A Computational Study. PhD Thesis.

[19] Irie M., Fukaminato T., Matsuda K., Kobatake S. (2014). Photochromism of diarylethene molecules and crystals: Memories, switches, and actuators. Chem. Rev..

[20] Ragazzon G., Baroncini M., Silvi S., Venturi M., Credi A. (2015). Light-powered autonomous and directional molecular motion of a dissipative self-assembling system. Nat. Nanotechnol..

[21] Merino E., Ribagorda M. (2012). Control over molecular motion using the cis-trans photoisomerization of the azo group. Beilstein J. Org. Chem..

[22] Oscurato S.L., Salvatore M., Maddalena P., Ambrosio A. (2018). From nanoscopic to macroscopic photo-driven motion in azobenzene-containing materials. Nanophotonics.

[23] Oscurato S.L., Borbone F., Maddalena P., Ambrosio A. (2017). Light-driven wettability tailoring of azopolymer surfaces with reconfigured three-dimensional posts. ACS Appl. Mater. Interfaces.

[24] Pirani F., Angelini A., Ricciardi S., Frascella F., Descrovi E. (2017). Laser-induced anisotropic wettability on azopolymeric micro-structures. Appl. Phys. Lett..

[25] Umlandt M., Kopyshev A., Pasechnik S.V., Zakharov A.V., Lomadze N., Santer S. (2022). Light-Triggered Manipulations of Droplets All in One: Reversible Wetting, Transport, Splitting, and Merging. ACS Appl. Mater. Interfaces.

[26] Wani O.M., Zeng H., Priimagi A. (2017). A light-driven artificial flytrap. Nat. Commun..

[27] Pagliusi P., Audia B., Provenzano C., Piñol M., Oriol L., Cipparrone G. (2019). Tunable Surface Patterning of Azopolymer by Vectorial Holography: The Role of Photoanisotropies in the Driving Force. ACS Appl. Mater. Interfaces.

[28] Priimagi A., Shevchenko A. (2014). Azopolymer-Based Micro- and Nanopatterning for Photonic Applications. J. Polym. Sci. Part B Polym. Phys..

[29] Hsu C., Xu Z., Wang X. (2018). Holographic Recording and Hierarchical Surface Patterning on Periodic Submicrometer Pillar Arrays of Azo Molecular Glass via Polarized Light Irradiation. Adv. Funct. Mater..

[30] Bagheri S., Giessen H., Neubrech F. (2014). Large-Area Antenna-Assisted SEIRA Substrates by Laser Interference Lithography. Adv. Opt. Mater..

[31] Reda F., Salvatore M., Borbone F., Maddalena P., Oscurato S.L. (2022). Accurate Morphology-Related Diffraction Behavior of Light-Induced Surface Relief Gratings on Azopolymers. ACS Mater. Lett..

[32] Rochon P., Batalla E., Natansohn A. (1995). Optically induced surface gratings on azoaromatic polymer-films. Appl. Phys. Lett..

[33] Kim D.Y., Tripathy S.K., Li L., Kumar J. (1995). Laser-induced holographic surface-relief gratings on nonlinear-optical polymer-films. Appl. Phys. Lett..

[34] Toshchevikov V., Saphiannikova M., Heinrich G. (2009). Microscopic Theory of Light-Induced Deformation in Amorphous Side-Chain Azobenzene Polymers. J. Phys. Chem. B.

[35] Toshchevikov V., Ilnytskyi J., Saphiannikova M. (2017). Photoisomerization Kinetics and Mechanical Stress in Azobenzene-Containing Materials. J. Phys. Chem. Lett..

[36] Toshchevikov V., Petrova T., Saphiannikova M. (2018). Kinetics of Ordering and Deformation in Photosensitive Azobenzene LC Networks. Polymers.

[37] Toshchevikov V., Saphiannikova M. (2023). Photo-Ordering and Deformation in Azobenzene-Containing Polymer Networks under Irradiation with Elliptically Polarized Light. Processes.

[38] Fuhrmann T., Tsutsui T. (1999). Synthesis and properties of a hole-conducting, photopatternable molecular glass. Chem. Mater..

[39] Shirota Y. (2005). Photo- and electroactive amorphous molecular materials—Molecular design, syntheses, reactions, properties, and applications. J. Mater. Chem..

[40] Saphiannikova M., Toshchevikov V. (2015). Optical deformations of azobenzene polymers: Orientation approach vs. photofluidization concept. J. Soc. Inf. Disp..

[41] Yadav B., Domurath J., Kim K., Lee S., Saphiannikova M. (2019). Orientation Approach to Directional Photodeformations in Glassy Side-Chain Azopolymers. J. Phys. Chem. B.

[42] Yadav B., Domurath J., Saphiannikova M. (2020). Modeling of Stripe Patterns in Photosensitive Azopolymers. Polymers.

[43] Loebner S., Yadav B., Lomadze N., Tverdokhleb N., Donner H., Saphiannikova M., Santer S. (2022). Local Direction of Optomechanical Stress in Azobenzene Containing Polymers During Surface Relief Grating Formation. Macromol. Mater. Eng..

[44] Holme N.C.R., Nikolova L., Ramanujam P.S., Hvilsted S. (1997). An analysis of the anisotropic and topographic gratings in a side-chain liquid crystalline azobenzene polyester. Appl. Phys. Lett..

[45] Viswanathan N.K., Balasubramanian S., Li L., Tripathy S.K., Kumar J. (1999). A detailed investigation of the polarization-dependent surface-relief-grating formation process on azo polymer films. Jpn. J. Appl. Phys. Part 1 Regul. Pap. Short Notes Rev. Pap..

[46] Lagugne-Labarthet F., Buffeteau T., Sourisseau C. (1999). Azopolymer holographic diffraction gratings: Time dependent analyses of the diffraction efficiency, birefringence, and surface modulation induced by two linearly polarized interfering beams. J. Phys. Chem. B.

[47] Rekola H., Berdin A., Fedele C., Virkki M., Priimagi A. (2020). Digital holographic microscopy for real-time observation of surface-relief grating formation on azobenzene-containing films. Sci. Rep..

[48] Sobolewska A., Bartkiewicz S. (2012). Surface relief grating in azo-polymer obtained for s-s polarization configuration of the writing beams. Appl. Phys. Lett..

[49] Yadavalli N.S., Santer S. (2013). In-situ atomic force microscopy study of the mechanism of surface relief grating formation in photosensitive polymer films. J. Appl. Phys..

[50] Yadavalli N.S., Loebner S., Papke T., Sava E., Hurduc N., Santer S. (2016). A comparative study of photoinduced deformation in azobenzene containing polymer films. Soft Matter.

[51] Kumar J., Li L., Jiang X.L., Kim D.Y., Lee T.S., Tripathy S. (1998). Gradient force: The mechanism for surface relief grating formation in azobenzene functionalized polymers. Appl. Phys. Lett..

[52] Viswanathan N.K., Kim D.Y., Bian S., Williams J., Liu W., Li L., Samuelson L., Kumar J., Tripathy S.K. (1999). Surface relief structures on azo polymer films. J. Mater. Chem..

[53] Bublitz D., Fleck B., Wenke L. (2001). A model for surface-relief formation in azobenzene polymers. Appl. Phys. B.

[54] Saphiannikova M., Neher D. (2005). Thermodynamic theory of light-induced material transport in amorphous azobenzene polymer films. J. Phys. Chem. B.

[55] Jelken J., Santer S. (2019). Light induced reversible structuring of photosensitive polymer films. RSC Adv..

[56] Fang G.J., Maclennan J.E., Yi Y., Glaser M.A., Farrow M., Korblova E., Walba D.M., Furtak T.E., Clark N.A. (2013). Athermal photofluidization of glasses. Nat. Commun..

