# Supplementary material for: Viscoplastic Modeling of Surface Relief Grating Growth on Isotropic and Preoriented Azopolymer Films
Source: Polymers (Basel). 2023 Jan 16;15(2):463. doi: 10.3390/polym15020463 (PMC9865333; doi:10.3390/polym15020463)
Supplement: Supplementary file 1 [file polymers-15-00463-s001.zip › SI_SRG_growth.pdf]

Supporting Information

Viscoplastic Modeling of Surface Relief Grating Growth on Isotropic and Pre-oriented Azopolymer Films

Nina Tverdokhleb,[1] Sarah Loebner,[2] Bharti Yadav,[1] Svetlana Santer,*[2] Marina Saphiannikova*[1]

[1] Institute Theory of Polymers, Leibniz Institute of Polymer Research Dresden, 01069 Dresden, Germany

[2] Institute of Physics and Astronomy, University of Potsdam, 14476 Potsdam, Germany

[*] Prof. Svetlana Santer,
e-mail: santer@uni-potsdam.de
University of Potsdam, Karl-Liebknecht Str. 24/25
14476 Potsdam, Germany

[*] Prof. Marina Saphiannikova,
e-mail: grenzer@ipfdd.de
Institute Theory of Polymers
Leibniz Institute of Polymer Research Dresden, Hohe Strasse 6
01069 Dresden, Germany

Table of contents

I. Inscription of surface relief gratings on initially isotropic sample 2

Influence of boundary conditions 2

Impact of Gaussian intensity distribution 5

Influence of the grating period *D* on the grating growth 7

II. Influence of initial orientation on phases of gratings 8

III. List of Videos 9

I. Inscription of surface relief gratings on initially isotropic sample

To determine the factors, that can influence the inscription of surface relief gratings from initial isotropic state, we model the parallelepiped samples with different boundary conditions (BCs) and various optical period *D*. Additionally we take into account the Gaussian intensity distribution of the laser beam.

Influence of boundary conditions (BCs)

On **Figures S1** and **S2** the initial samples have parallelepiped shape with size $6\times10\times1\ \mu m^3$. Rotational and translational movement of the sample is prohibited by “gluing” its bottom surface to the substrate. The upper surface is left free. The BCs along the grating vector (x-axis) correspond to the “Frictionless support” in ANSYS, which restricts the movements perpendicular to x-axis. Changing BCs in y-direction gives different appearance of gratings under irradiation with PP, SS and RL IPs. So, at free BCs without restrictions along y-axis (**Figure S1**), all IPs produce protrusions at the grating edges. Due to elongation along y-axis, a very weak SS grating of 2 nm height can be inscribed. BCs imposed by Frictionless support in y-direction (**Figure S2**) totally supress the inscription of SS grating: the sample surface does not deform. Frictionless support in y-direction leads to obscure structures at the edges of topographical gratings inscribed by the RL IP that obstruct a further analysis.

The periodic BCs in y-direction allow us to model SRGs with symmetric appearance, as it is observed in experiments. This case is chosen for the analysis of grating growth in the main text. As discussed there, SS gratings cannot be inscribed from initial isotropic state of polymer backbones at periodic BCs.

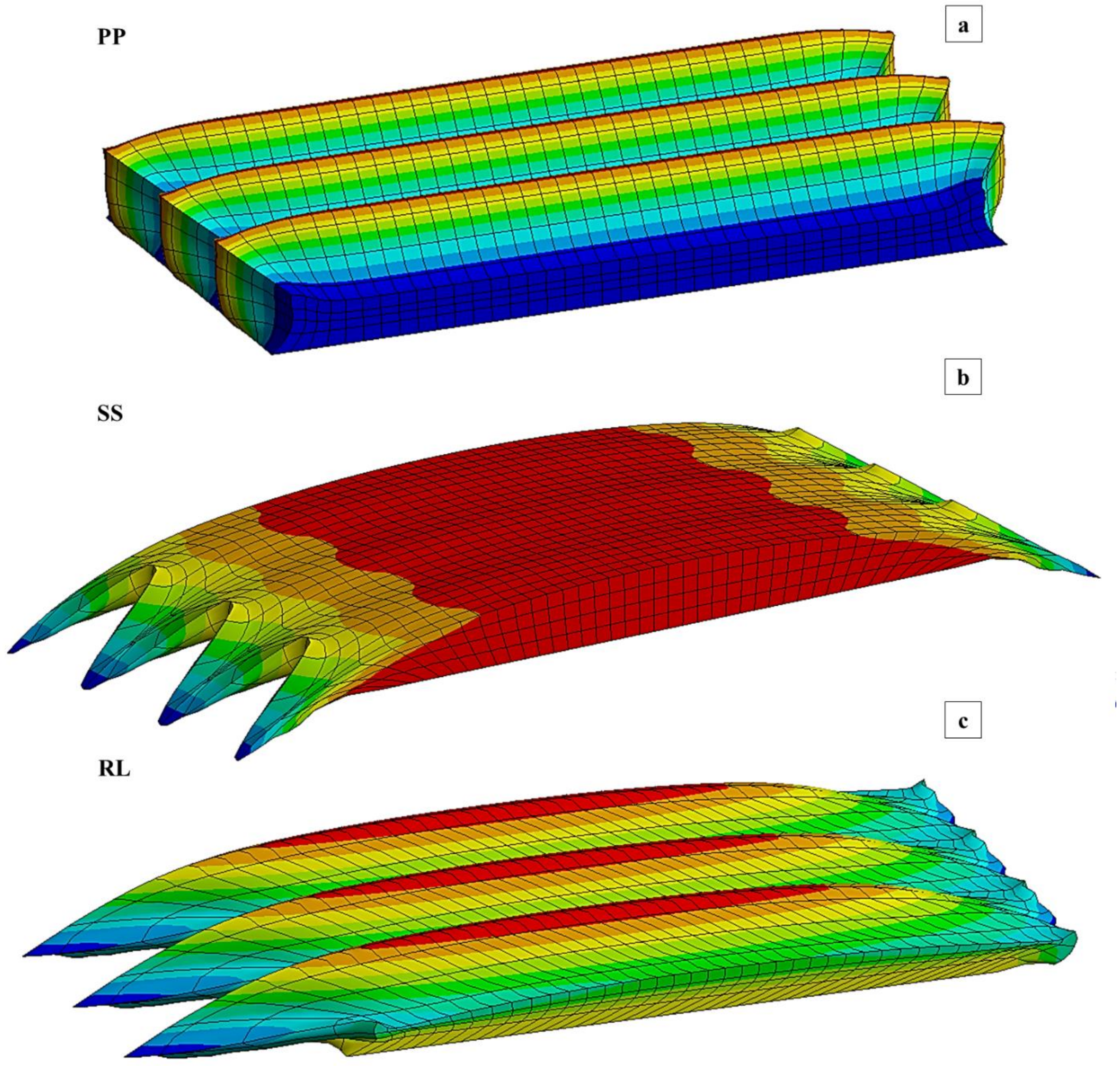

Figure S1. Modeled gratings with free BCs in y-direction after 100 s of inscription with PP, SS and RL interference patterns. Initial isotropic state. Colors correspond to directional deformations along z-axis: from maximal stretching (red) to maximal compression (blue).

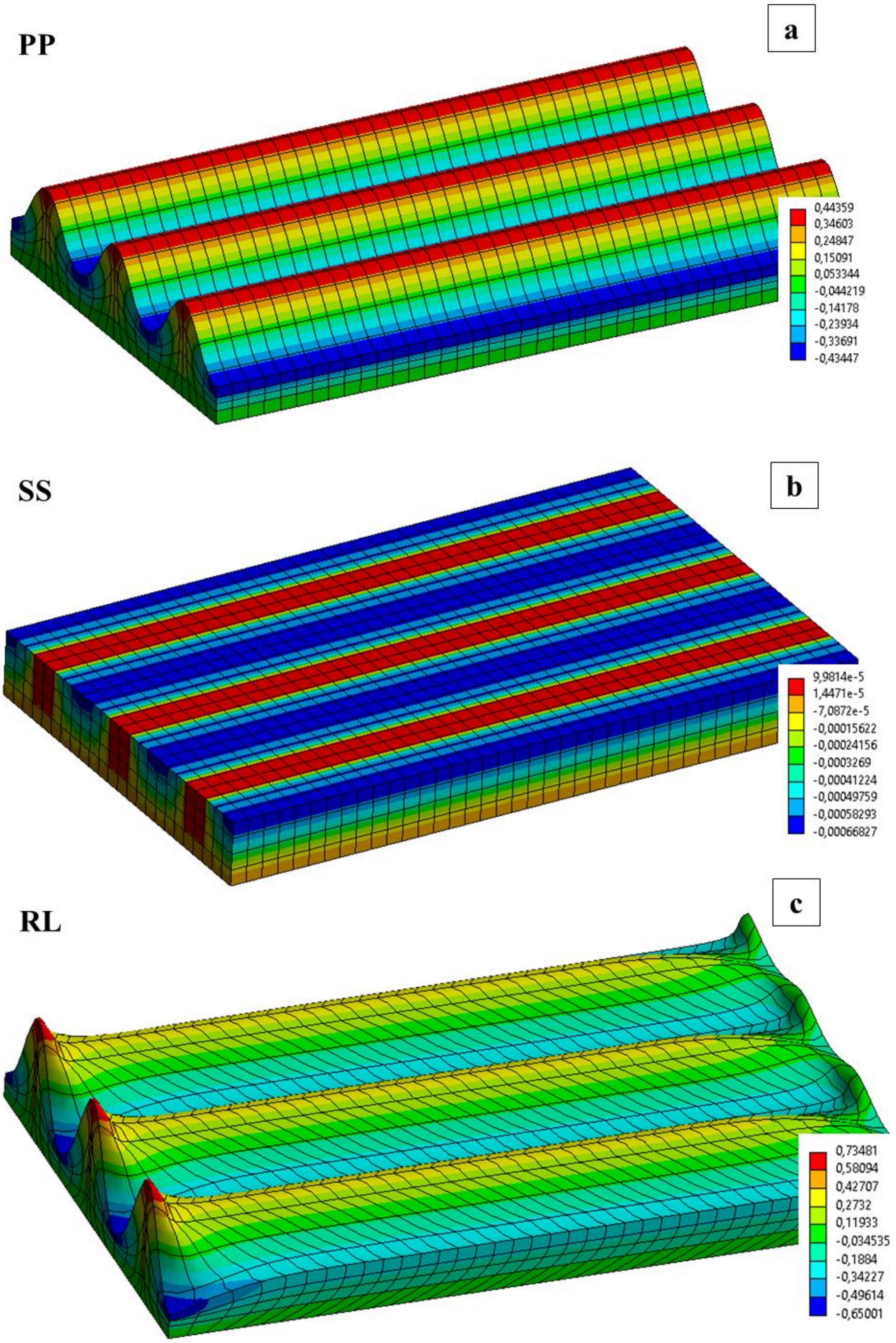

Figure S2. Modeled gratings with Frictionless support in y-direction after 100 s of inscription with PP, SS and RL interference patterns. Initial isotropic state. Colors correspond to directional deformations along z-axis: from maximal stretching (red) to maximal compression (blue).

Impact of Gaussian intensity distribution

To check the influence of the Gaussian distribution on the efficiency of gratings inscribed from initial isotropic state, we multiply eq. (4) in the main text on the factor $\exp\left(-\frac{2y^2}{\omega^2}\right)$, where y is the y-coordinate of central position of the elements in the modelled sample, ω is the radius of Gaussian laser beam. Only for a highly focused beam with $\omega = 1$ μm we receive visible surface deformations under SS IP (**Figure S3a**), but their shape does not correspond to the sinusoidal gratings. With the increase of ω to 10 μm (**Figure S3b**), deformations become negligible. This is far below the size of irradiated spot in the experiment, where $\omega \approx 2$ mm.

As for PP IP produced by a strongly focused Gaussian beam, $\omega = 5$ μm, the height of the grating decays from the center of the irradiated spot to its edges (**Figure S3c**). The appearance of RL grating under strongly focused beam is smoothed at the edges, its overall height decreases (**Figure S3d**). For the Gaussian beams with $\omega \geq 100$ μm, the height of PP and RL gratings becomes close to that calculated neglecting the Gaussian intensity distribution.

To summarize, the height and appearance of gratings is not influenced by the intensity distribution in the irradiated spot, when its size is taken close to the experimental one.

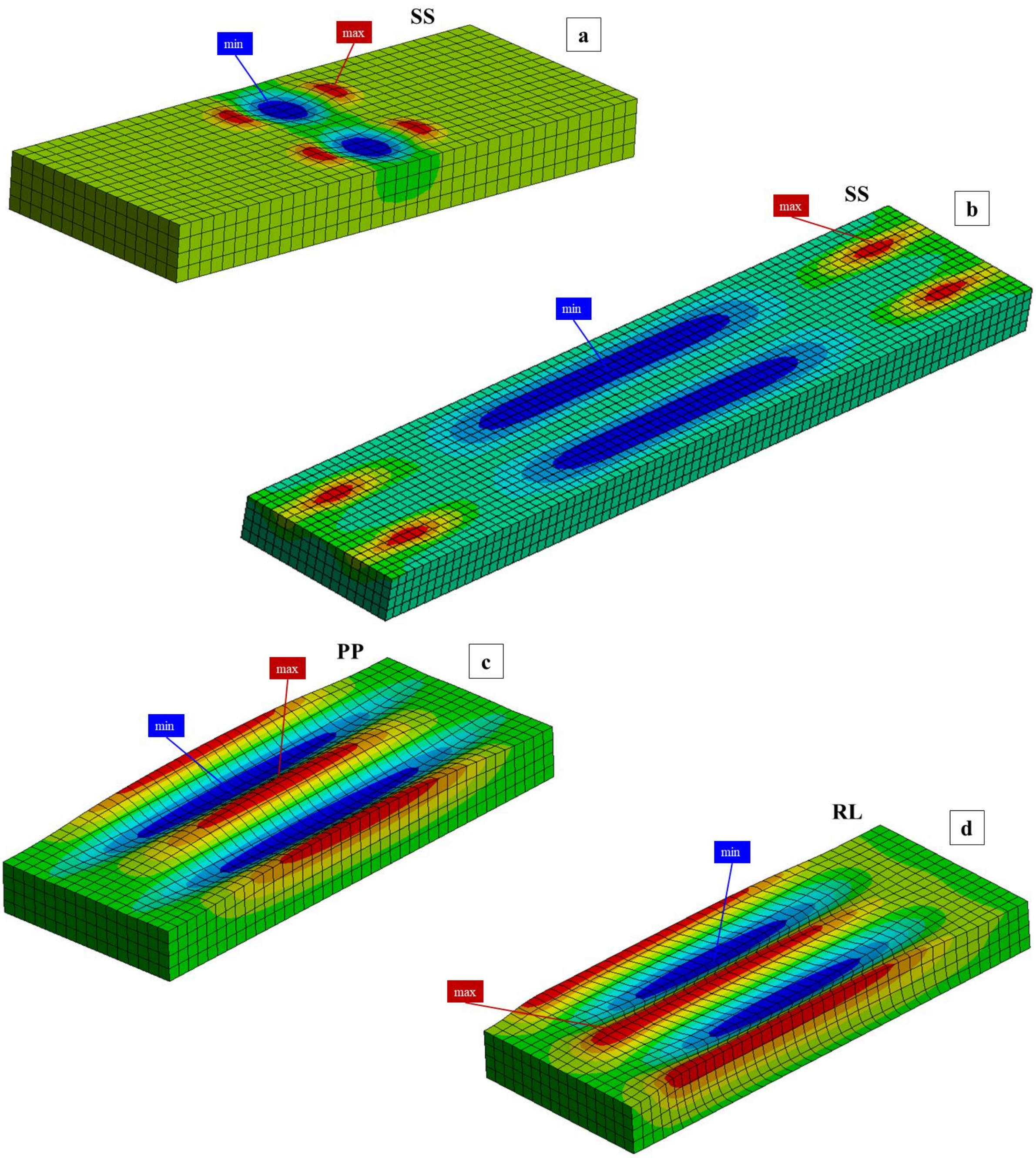

Figure S3. Modeled gratings with Frictionless support in y-direction after 100 s of inscription by differently focused Gaussian beams: **a)** SS, $\omega = 1\ \mu$m, **b)** SS, $\omega = 10\ \mu$m, **c)** PP, $\omega = 5\ \mu$m, **d)** RL, $\omega = 5\ \mu$m. The size of the sample in **a**, **c** and **d** is $4\times10\times1\ \mu m^3$, for **b** the size is taken $4\times20\times1\ \mu m^3$ to accommodate a larger irradiation spot. Initial isotropic state.

Influence of the grating period D on the grating growth

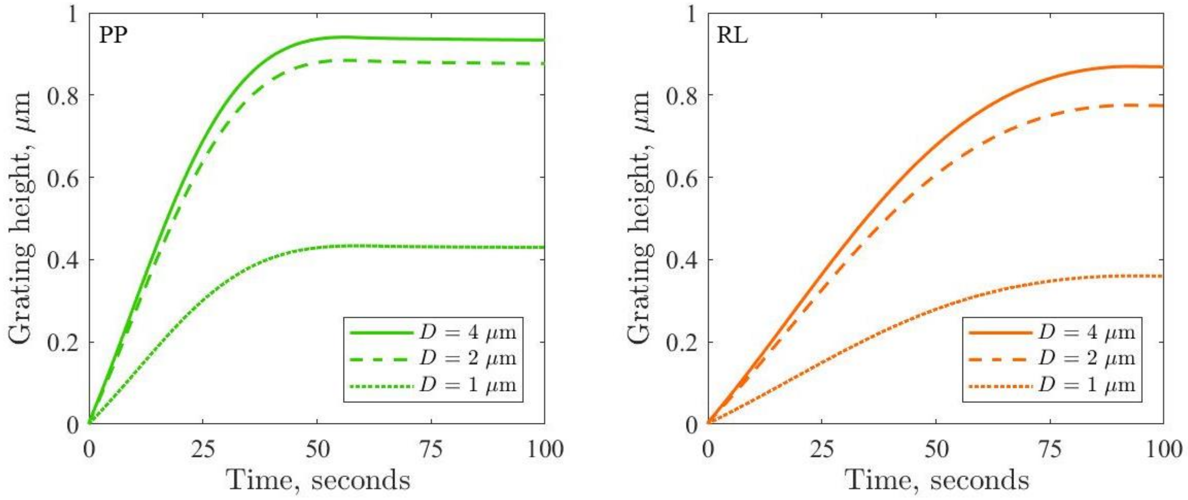

Figure S4. Time-dependent growth of PP and RL gratings at different optical periods: D = 1, 2 and 4 μm. Initial isotropic state. Note that the height of both gratings increases with the grating period.

II. The Influence of initial orientation on phases of gratings

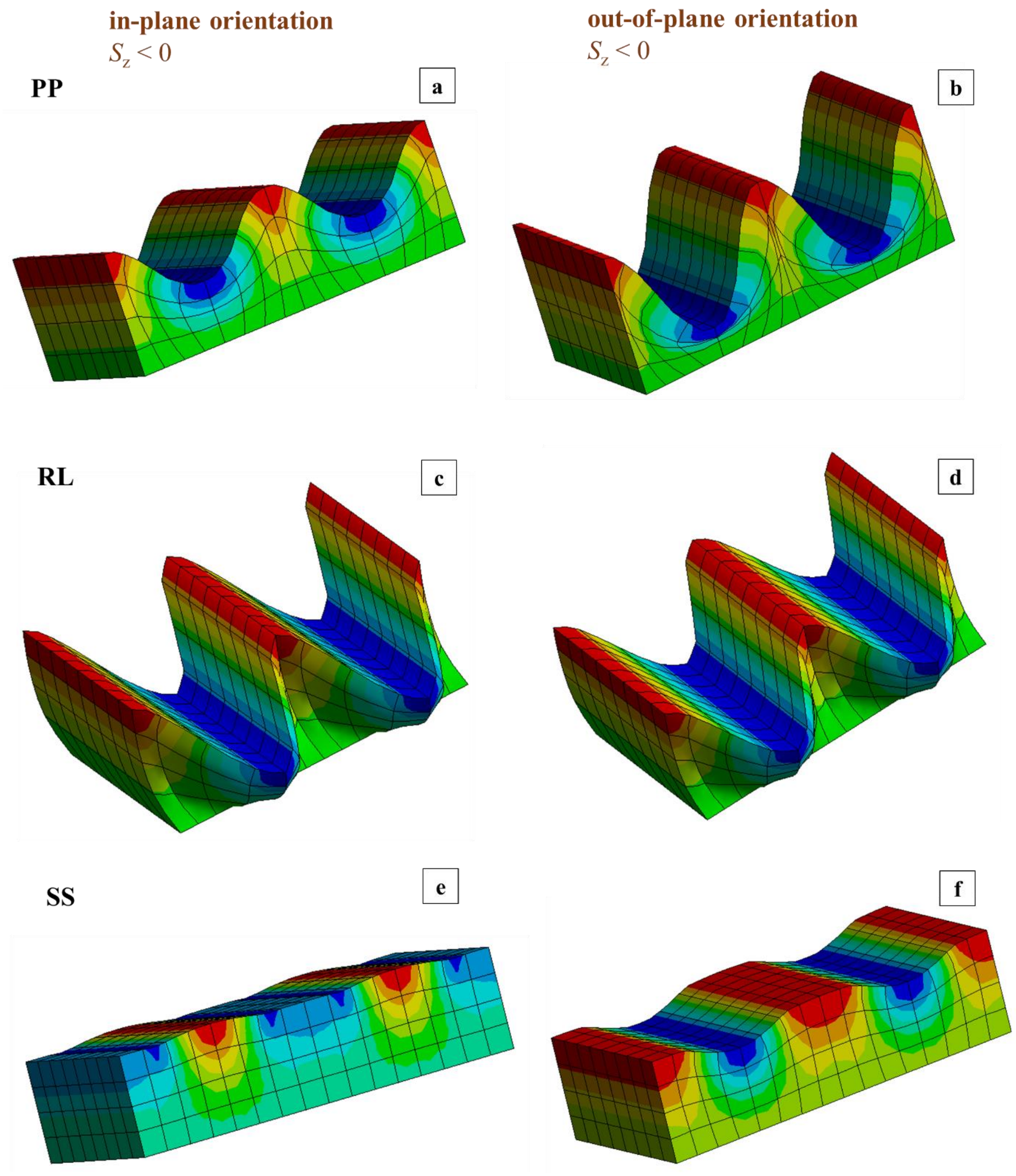

Figure S5. Modeled gratings for two initial anisotropic cases discussed in the main text. **a**, **c**, **e** – case 1 and **b**, **d**, **f** – case 2. Infinite sample with periodic BCs in y-direction has the unit cell $4{\times}2{\times}1\ \mu m^3$. Note that the phase of PP and RL gratings is not influenced by the initial orientation state, whereas the phase of SS grating flips: the maximal stretching along z-axis (red hills) grow either at maximal or minimal intensity of light.

III. List of Videos

Parameters for all modeled **samples:** size 6×10×1 μm^3, τ_{yield} =10 MPa, γ = 0.01 s^{-1} and λ = 1000 s; initial light-induced stress $\tau_{light,0} = 25$ MPa. Colors correspond to directional deformations along z-axis: from maximal stretching (red) to maximal compression (blue).

Video S1 shows a plastic deformation of the azopolymer film under irradiation with SS interference pattern from *initial isotropic state*.

Video S2 shows a plastic deformation of the azopolymer film under irradiation with SS interference pattern from *in-plane orientation* of polymer backbones (case 1).

Video S3 shows a plastic deformation of the azopolymer film under irradiation with SS interference pattern from *out-of-plane orientation* of polymer backbones (case 2).
